# Uncommon cause of small bowel obstruction - gallstone ileus: a case report

**DOI:** 10.1186/1757-1626-2-9321

**Published:** 2009-12-14

**Authors:** Fatima Ezzahra Zahid, El Bachir Benjelloun, Abdelmalek Ousadden, Khalid Mazaz, Khalid Ait Taleb

**Affiliations:** 1 Department of general surgery, University hospital Hassan II, Fes, Morocco

## Abstract

Gallstone ileus is an uncommon cause of small bowel obstruction. We present a case of small intestinal obstruction owing to a large gallstone in lower ileum in a 65 years old man. The diagnosis was made by computed tomography.

## Background

Gallstone ileus is an uncommon condition that may result when a gallbladder stone enters into the intestinal tract, usually as a result of an internal fistula between the gallbladder and the duodenum. It's accounting for only 1-4% of all intestinal obstruction [1]. In patient with cholilithiasis only 0.3-0.5% develop gallstone ileus [2]. The mortality reportedly ranges from 12-18%, particularly in older patient who often have co morbid illness [3]. We reported a case of a 65 years old man, who present with signs of small bowel obstruction, owing to a large gallstone in lower ileum.

## Case Presentation

A 65-year old previously healthy man, presented to the Emergency Department, with complaints of abdominal pain, vomiting and absolute constipation of 2 days duration. The patient was not using any specific medication and his medical history did not suggest a major disease. He had no prior history of abdominal surgery or trauma. The patient didn't smoke or drink alcohol.

**Figure 1:**
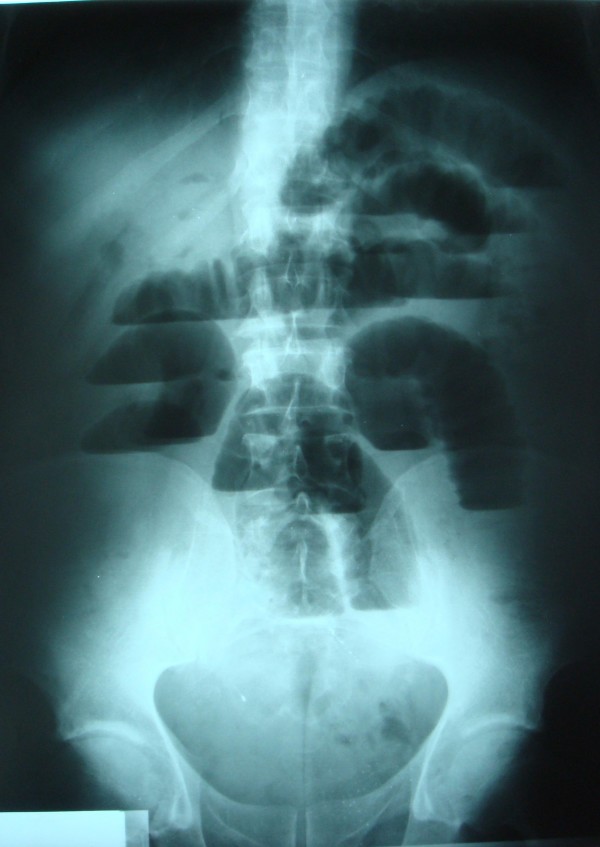
**Abdominal X ray shows air fluid levels**.

**Figure 2:**
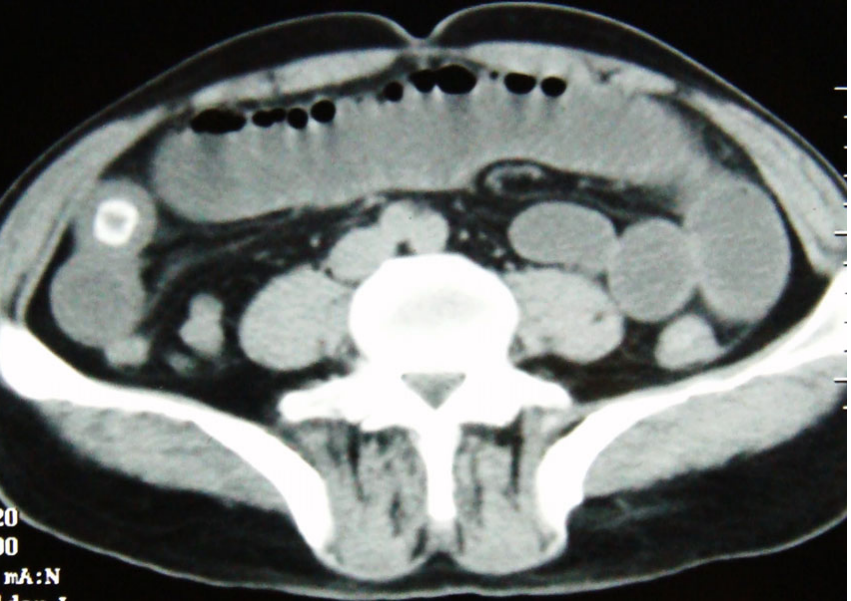
**Abdominal tomography shows a calcified mass in lower ileum**.

**Figure 3:**
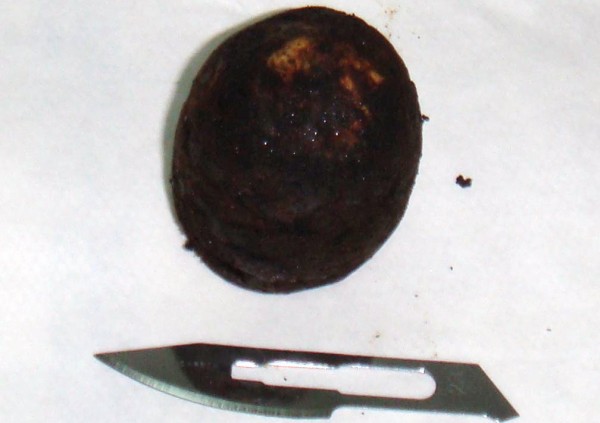
**Gallstone extracted from the ileum**.

Physical examination revealed conscious dehydrated patient; vital signs were within normal limits. The temperature was of 37°C, a pulse rate 100 beat per minute (bpm), a blood pressure 12/07 mm Hg. Abdominal examination reveals distend abdomen without tender. There were no palpable masses or liver enlargement. The hernial sites were free. On rectal examination there is no stool. Laboratory examination showed hemoglobin of 14 g/dl, leucocytes of 12000 cells per cubic millimeter, blood urea 0.8 g/dl, and a normal liver function profile. An abdominal X-ray showed small bowel air-fluid levels (fig 1). A nasogastric tube was placed with return of 2 L bilious fluid. Beside abdominal ultrasonography was performed demonstrating an enlarged loops of small bowel, and scleroatrophique gallbladder with gallstone. An abdominal tomography showed distended small bowel loops secondary to a laminated calcified mass in the lower ileum that suggested a gallstone ileus (fig 2). After proper optimization, the patient was taken up for surgery. At exploration, the peritoneal cavity was filled with 300 cc of free serosal fluid, while numerous dilated loops of small bowel. A 4 cm gallstone was found obstructing the distal ileum 3 cm before Bauhin valve (fig 3). The gallstone was extracted by means of a longitudinal enterotomy, which was then closed transversally. The gallbladder was surrounded by an intense inflammatory reaction. The plan is for the patient to return for cholecystectomy and fistula repair at a later date. The follow up was simple.

## Discussion

Gallstone ileus is an uncommon cause of small bowel obstruction [1-6]. It occurs almost exclusively in the elderly, and account for 25% of mechanical small-bowel obstruction in patient over the age of 65, with a mortality of 12-50% [4]. This pathology occurs three to five times more frequently in women than in men [2]. The gallstone enters the intestinal tract through a fistula formed between the gallbladder and the duodenum, stomach or colon. In particular, a cholecystodudenal fistula was identified in 68% of patients with gallstone ileus [6]. The terminal ileum is the most frequent site of obstruction [1]. However, it may be found in the duodenum causing Bouveret's syndrome [2]. Other obstruction points, including jejunum (30%) and colon (2.5%) may be seen. Plain abdominal radiographs may reveal signs of small bowel obstruction and concomitant aerobilia to suggest the diagnosis [7,8]. The classical radiologic triad or Rigler triad of pneumobilia, small bowel obstruction and ectopic gallstone is specific for this disease, but only 9-14% of patients have a clear-cut Rigler's triad [7]. Computed tomography is the investigation of choice. The principal goal in management of gallstone ileus is a quick effective relief of mechanical bowel obstruction. Spontaneous passage of gallstones large enough to cause impaction has been reported, but most patients require intervention. If the stone is in within reach of an endoscope, either in the proximal small bowel or in the colon, it may be treated by lithotripsy and removal of the fragment[2]. Extracorporeal shockwave lithotripsy has also been used successfully, but this method is limitedby bowel gas. Unfortunately, the majority of patients require surgery. Surgical options include enterotomy and removal of the stones (enterolithotomy), enterolithotomy plus cholecystectomy and repair of the fistula [3]. Most authors favor enterolithotomy alone, followed by cholecystectomy at later date, because of its lower morbidity and report high spontaneous fistula closure up to 50% [7].

## Conclusion

Although it's a rare cause of bowel obstruction, gallstone ileus should be capped in mind when dealing with a case of small bowel obstruction, especially in elderly patient in whom the diagnosis is easily ignored. Early surgical intervention is the mainstay of treatment.

## Consent

Written informed consent was obtained for publication of this case report and accompanying images. A copy of the written consent is available for review by the Editor-in-Chief of this journal.

## Competing interests

The authors declare that they have no competing interests.

## Authors' contributions

FZ is a surgeon who was drafting the manuscript and revising it critically for content. EB is a surgeon who was involved in literature research. KM, KA were surgeons treating of the patient and were involved in revising the draft critically for content. AO is a surgeon was getting photographs and was involved in drafting manuscript. All authors have given final approval of the revision to be published.
